# Autism data sharing: Benefits, challenges, and recommendations

**DOI:** 10.1371/journal.pdig.0001249

**Published:** 2026-03-02

**Authors:** Alexandra Lautarescu, Brett Trost, Azadeh Kushki, Bethany Oakley, Síofra Heraty, David Belton, Alison Boyle, Sarah Douglas, Ciara J. Molloy, Rosemary Holt, Madeleine Bloomfield, Florence Campana, Miro Cupak, Erica Stevenson, Julian Tillmann, Christopher Chatham, Evdokia Anagnostou, Dean Hartley, Tony Charman

**Affiliations:** 1 Department of Psychology, Institute of Psychiatry, Psychology & Neuroscience, King’s College London, London, United Kingdom; 2 Department of Forensic and Neurodevelopmental Sciences, Institute of Psychiatry, Psychology & Neuroscience, King’s College London, London, United Kingdom; 3 Programs in Molecular Medicine and Genetics & Genome Biology, The Hospital for Sick Children, Toronto, Ontario, Canada; 4 Holland Bloorview Kids Rehabilitation Hospital, Toronto, Ontario, Canada; 5 Institute of Biomedical Engineering, University of Toronto, Ontario, Canada; 6 Department of Psychological Sciences, Birkbeck, University of London, London, United Kingdom; 7 AIMS-2-Trials Autism Representatives, University of Cambridge, Cambridge, United Kingdom; 8 Department of Psychiatry, School of Medicine, Trinity College Dublin, Dublin, Ireland; 9 Autism Research Centre, Department of Psychiatry, University of Cambridge, Cambridge, United Kingdom; 10 Department of Psychological Medicine, Institute of Psychiatry, Psychology & Neuroscience, King’s College London, London, United Kingdom; 11 Institut Pasteur, Paris, France; 12 DNAstack, Toronto, Ontario, Canada; 13 Province of Ontario Neurodevelopmental Network Autism Representative, Toronto, Ontario, Canada; 14 F. Hoffman-La Roche Ltd, Basel, Switzerland; 15 Department of Pediatrics, University of Toronto, Toronto, Ontario, Canada; 16 Autism Speaks, Washington, D.C., United States of America; CHOP: The Children's Hospital of Philadelphia, UNITED STATES OF AMERICA

## Abstract

Data sharing is a key element of scientific research, but it is associated with many complex legal, ethical, and practical challenges. These are particularly salient in autism research, where concerns have been raised about researchers’ intentions, research priorities not aligning with those of autistic people, and differing opinions within stakeholder communities as to what priorities should be addressed. This review paper was co-produced through an iterative collaborative process to incorporate diverse viewpoints of stakeholder representatives from academia, charity, industry, the medical community, and the autism community. We discuss the main benefits and challenges of autism data sharing and argue that the perspectives of autistic people must be central to discussions around its ethical and technological aspects. We outline recommendations for ethical and responsible data sharing practices and note key developments within the field, including federated data sharing and community platforms and registries.

## Author summary

Data sharing is becoming more important in scientific research. Larger datasets enable more detailed and accurate information to be analysed and can lead to better collaboration among researchers. In this review, we look at the main benefits and challenges of data sharing in autism research, where people have raised concerns around data sharing. We discuss the importance of including autistic people in all discussions about data sharing. Based on discussions between representatives from different stakeholder communities, we recommend practices that could make data sharing more ethical and responsible. We conclude the review by outlining recent developments in this area and discussing their potential implications for autism data sharing.

## 1. Introduction

### 1.1. Background

Addressing complex scientific questions in a reliable and replicable way often requires large amounts of data, which can be slow and expensive to collect, and thus is limited to a small number of well-funded researchers/consortia. For autism research, large datasets are needed to adequately represent the variability in the autism spectrum and allow for sufficient statistical power to answer critical research questions that aim to improve outcomes for autistic people (e.g., through improved understanding of developmental processes and trajectories across the lifespan, and provision of more effective, tailored support and services to enhance mental/physical health). To address this, data sharing is becoming an integral part of the research landscape [1–3], though it is a broad term that encompasses a range of approaches utilised in current practice. For instance, levels of data access among existing data sharing models can include: public data sharing (without restrictions), registered-access sharing (with approved users), controlled-access sharing (with approved users on a project-by-project basis), clique sharing (e.g., within a consortium), or sharing upon request (not usually shared in practice) [4–6]. Access to data related to neurodevelopmental/neuropsychiatric conditions (including autism) varies across these levels, even within similar data types (e.g., neuroimaging research, see [7]).

Data sharing is challenging; data are often spread out among multiple datasets and cohorts worldwide, with differences in physical locations, data acquisition methods, and legal and regulatory requirements for data access. Autism research data are often shared through initiatives that are focussed on autism and/or other neurodevelopmental conditions (see Table 1, part A), but research data from autistic participants are also shared through initiatives with a wider focus (see Table 1, part B).

**Table 1:** Examples of data sharing initiatives that include autism data.

Initiative	Link
**Part A. Autism Data Sharing Initiatives**	
The Simons Foundation – Simons Simplex Collection	https://www.sfari.org/resource/simons-simplex-collection [8]
The Simons Foundation – SPARK	https://sparkforautism.org/ [9]
The Simons Foundation – Searchlight	https://www.simonssearchlight.org/ [10]
Autism Speaks – Autism Genetic Resource Exchange (AGRE)	https://www.autismspeaks.org/agre [11]
Autism Speaks – MSSNG	https://research.mss.ng/ [12]
The Autism Sequencing Consortium	https://asc.broadinstitute.org/ [13]
Autism Brain Imaging Data Exchange I and II (ABIDE)	https://fcon_1000.projects.nitrc.org/indi/abide/ [14]
The iTARGET project	https://www.itargetautism.ca/ [15]
**Part B. Initiatives with a wider focus**	
Psychiatric Genomics Consortium	https://pgc.unc.edu/ [16]
The National Human Genome Research Institute AnVIL	https://anvilproject.org/ [17]
National Institute of Mental Health (NIMH) Data Archive (NDA)	https://nda.nih.gov [18]
The European Genome-Phenome Archive	https://ega-archive.org/ [19]
ELIXIR	https://elixir-europe.org/ [20]
iPsych	https://ipsych.dk/en [21]

Regulatory and legislative frameworks that govern data sharing policy and practice are complex to navigate, change over time, and can be interpreted differently by individual academics, consortia, and institutions. Examples include the HHS Office of Human Research Protections (OHRP) regulations [22] and the NIH Data Management and Sharing Policy [23,24] in the United States and the General Data Protection Regulation (GDPR) [25] in the European Union. Perceptions on data sharing may also differ between countries [26–29].

For autism research, public unrestricted data sharing is rare (e.g., the metadata from the “Development of Robot-Enhanced therapy for children with Autism spectrum disorders” [30]); access to most autism datasets requires steps that range from relatively straightforward online applications (e.g., Simons Searchlight, [10]) to extensive data sharing agreements (e.g., MSSNG, [12]). This complexity has led some participant community members to develop their own independent data sharing initiatives (e.g., through https://www.citizen.health/, [31]). Further, the costs associated with data sharing (e.g., to cover data collation and curation in increasingly complex, multimodal, and multisite projects) are often a barrier to sustainability, particularly in low-resource settings. This may lead to a more widespread use of national and regional registries and biobanks, with global initiatives to link these together, such as the Global Biobank Meta-analysis initiative (https://www.globalbiobankmeta.org/ [32]).

In addition, concerns about the direction of autism research and how data are shared, safeguarded, and interpreted [33] have been raised by the autism community [34,35]. A salient example is Spectrum 10K (https://spectrum10k.org/, [36]), a UK study investigating autism genetics, which was closed due to concerns from autistic people around the potential for its data to be used for eugenics, prenatal screening, and autism cures [37].

In this review, we conceptualise data sharing as a potential amplifier of both negative and positive aspects of autism research, outline its core benefits and challenges, and provide recommendations for best practices. We argue that to streamline and safeguard access to data and encourage more research that can serve the needs of the autism community, it is imperative to develop a unified legal, ethical, and technological framework. The launch of new collaborations and data sharing initiatives within the autism research sphere (e.g., Autism Sharing Initiative, ASI, [38,39]) provides an ideal opportunity for reflection and scope-setting. It is essential that such discussions include the diverse perspectives of multiple stakeholder communities (i.e., including academia and professionals working with autistic people such as clinicians, charities, and industry) and centre the views of those impacted by the research (i.e., autistic people and their families) [40,41]. There must be an understanding that perspectives may differ not only between stakeholders, but also depending on contextual factors such as the type of research being conducted [42,43] and geographical location [44].

### 1.2. Review-writing process

This manuscript is based on work done as part of the Autism Innovative Medicine Studies-2-Trials (AIMS-2-TRIALS) consortium (aims-2-trials.eu, [45]), an autism biomedical research programme. In AIMS-2-TRIALS, we identified key infrastructural/procedural requirements and developed principles and policies for data sharing that aim to address and mitigate risks and concerns. Many of these strategies also have wider applicability for the autism research community. This motivated us to write the current review as a co-produced, collaborative exercise to incorporate diverse viewpoints of stakeholder representatives.

This review was written by a team of autistic and non-autistic authors, including those from academia, charity, industry, the medical community, and the autism community. Co-authors were invited to contribute to this review based on their roles in key collaborative activities led by the AIMS-2-TRIALS consortium. Members of the autism community (i.e., autistic people and family members or carers of autistic people) were included through their roles as AIMS-2-TRIALS Autism Representatives (A-Reps) and Province of Ontario Neurodevelopmental Disorders (POND; https://pond-network.ca/, [46]) Representatives. As part of AIMS-2-TRIALS work, data sharing was discussed in depth as part of several working groups: Ethics and Data Sharing, ASI and Sustainability, and European Autism GEnomics Registry (EAGER) [47]. Input was given either directly as part of regular online meetings or occasional in-person meetings (e.g., as part of the AIMS-2-TRIALS Annual General Meetings), or through communication via email or contributions to collaborative documents. A total of 30 meetings were organised across these working groups, and the need to write the current review emerged as a result of these discussions. Co-authors from charity, industry, and the medical community were included based on collaborative activities between AIMS-2-TRIALS [45], and North American colleagues from the POND network [46], and MSSNG cohorts [12]. Draft sections were iteratively developed in shared online documents, with all co-authors invited to comment and propose revisions. Divergent viewpoints were discussed during group meetings until consensus was reached, ensuring that the final text reflected a balanced representation of stakeholder perspectives. The writing process was collaborative, with all authors contributing to identifying key topics, refining arguments, and incorporating relevant literature to support the topics and perspectives highlighted by the group.

## 2. How can autism data sharing benefit the autism community?

Many autistic people are underserved, with inadequate access to services and support, and may be more vulnerable to poor or inappropriate advice (see panel 7 in [48]). Questions of value to autistic people and their families [48] often cannot be answered reliably with the low statistical power in neurodevelopmental research [35,49,50]. Responsible data sharing can address this challenge by improving the quantity, quality, utility, and reliability of research (see **Box** 1) and, when linked to research priorities identified by the autistic community, can lead to a better understanding of autism and a better quality of life for autistic people across the lifespan [51].

Box 1. Benefits of data sharing*

- Aligns with FAIR principles for research data: Findability, Accessibility, Interoperability, and Reuse [52]
- Encourages openness and accountability and reduces the potential for data to be fabricated, falsified, or distorted
- Encourages researchers to replicate scientific findings in other datasets/cohorts
- Allows analyses to be performed on larger sample sizes, increasing statistical power and promoting greater confidence in scientific findings
- Promotes collaboration and facilitates multidisciplinary research, as data are equally accessible to all (unless access is cost-prohibitive)
- Makes data accessible to a larger and more diverse set of researchers, in particular those who lack the resources to generate similar data themselves
- Facilitates data re-analysis with new or more advanced techniques than those available when the data were generated
- Facilitates data aggregation by researchers who are not experts in a particular discipline/technique and could not pre-process or process the raw data
- Accelerates research findings (e.g., [53])
- Reduces costs through the reuse of data
- Increases data longevity
- Improves data management and safety protocols (when shared through platforms that have standards in place to incentivise such behaviour)
- Reduces burden on participants who may otherwise be recruited to multiple projects that are addressing similar issues using similar tools.

*Adapted from Levenstein and Lyle [54] and Munafò and colleagues [55].

### 2.1. Gaining a better understanding of autism

Decades of research have resulted in only a limited understanding of the factors underpinning many of the key characteristics exhibited by autistic people, including biological (e.g., biomarkers, rare genetic variants, and common polygenic variation), phenotypic (e.g., sensory differences), and environmental factors (e.g., societal barriers). Data sharing can improve our understanding of these factors [56], informing interventions, supports, and societal changes that can improve the quality of life of autistic people [57], such as strategies for alleviating sleep difficulties [58].

A better understanding of autism will be made possible through analysis of larger or pooled datasets, which can increase prediction utility [57,59] and reveal patterns not apparent in smaller datasets [60,61], as demonstrated in other fields [62,63]. This will also enable replication [64] and allow researchers to assess complex multifactorial influences; something that currently only a few groups can do with their moderately sized cohorts (e.g., POND [46], AIMS-2-TRIALS [45], Simons Simplex Collection [8], MSSNG [12]).

Data sharing can thus help move autism research away from solely binary comparisons (e.g., autistic versus neurotypical participants [65]) to more nuanced comparisons, such as ‘normative modelling’ approaches, which enable us to understand differences at the level of individual participants by comparing their data to the expected pattern in a large reference sample [66,67]. Additional approaches may involve comparing participants with varied neurodevelopmental conditions/neurodivergent differences, or subsetting autistic participants based on genetic or phenotypic characteristics, which may yield patterns not apparent when considering a heterogenous group of autistic people as a whole [68,69]. This can, in turn, enable the identification of groups of autistic people who have similar characteristics and who may want, and benefit from, particular interventions or care pathways.

### 2.2. Promoting more ethical, transparent, and inclusive autism research

Currently, much of biomedical and psychological research is not reproducible, fuelling recent calls for increased research transparency, integrity, and openness [55]. Reproducibility of autism research can be hampered by various issues, including the combination of small effect sizes and sampling variability [70] and a lack of transparency regarding what and how data are analysed (e.g., [71,72]).

In the context of recent calls for incorporating open science methods in autism research [35,73], data sharing has the potential to improve research quality and accelerate the pace of research in an ethical manner [4]. For example, sharing within a consortium (e.g., AIMS-2-TRIALS [45], POND [46]) enables collaborative work by bringing together different data, approaches, expertise, and interpretation.

When compiled from sufficiently diverse sources, data sharing can enable greater representativeness in autism research cohorts. Meaningful inclusion of diverse identities is essential but presently overlooked [74], with many groups being underrepresented in autism research, including (but not limited to) autistic people who are non-speaking, have an intellectual disability, are from non-White ethnic minority backgrounds, live outside of the Global North, or people who do not have access to a diagnosis or do not meet the current threshold for diagnosis (e.g., those with “subclinical” presentations, sometimes called the broader autism phenotype) [75,76]. Data sharing has the potential to better reveal the individuality of autistic people based not only on their biological and phenotypic characteristics but also their sociological and intersectional identities. However, in the Global South, gathering and sharing data has particular challenges including limited resources and infrastructure [29,77], and inequities between researchers in different countries and between researchers and communities [78–80]. Ensuring digital inclusion [81] is largely dependent on increased support from funders (e.g., Wellcome Trust support for the iSHARE2 project; http://www.indepth-ishare.org/ [82]). To ensure that autistic people who live in low-resource settings are appropriately represented in research efforts, researchers from the Global South require better access to data storage and analytical infrastructure, practical frameworks for data sharing across different contexts, co-leadership in data sharing discussions, and opportunities for autism researchers to publish findings rather than being constrained to the role of data production [78,79,83].

The benefits of data sharing can only be fully realised if the entire stakeholder community is included in the research process [84]. There is an urgent need for the autistic community to be included in the research process beyond just being “data donors”, with calls for true participatory research or “co-design” being at the forefront of discussions [85]. In the future, this could improve the quality and richness of the data not only for the researchers, but for the autism community to share amongst themselves. Data sharing can create a cyclical benefit, wherein greater collaboration with the autistic community encourages greater participation in research by autistic people, which in turn increases the size of datasets and the efficacy and quality of analyses.

## 3. What are the risks of data sharing for autism research, and how can we minimise them?

Approaches to autism data sharing must align with principles of data justice and data solidarity [86], with considerations for who benefits from the data, who bears the risks (and how these can be reduced, and whether data management and sharing practices reinforce or reduce inequalities. Responsible data sharing requires participants and/ or their carers/ legal guardians to be fully informed of any known or potential risks [87]. Data collection generally occurs over a specific time period, whereas risk to both individual participants and the groups they represent extends past the collection period and may fluctuate based on the research, clinical, and societal climate at any given time. Autistic people can be vulnerable to stigma and bias that could be exacerbated by irresponsible data handling. Discussions about autism data sharing must also include careful consideration of the inclusion of autistic children and adults with high support needs, who may have someone else consenting on their behalf [88–90].

Currently, there is a paucity of autism-specific research addressing community views on data sharing. While many participants are supportive of data sharing if appropriately informed [91], common concerns include those around unintended use of data, biased data interpretation, and security (e.g., confidentiality, re-identification). We outline below key potential risks and suggest how they could be minimised. We argue that open and reproducible science and participatory research practices both can and should work together, and that creative solutions from this intersection will benefit both researchers and the communities they serve. Key risks and potential solutions are summarised in Table 2 and expanded upon in the sections below.

**Table 2:** Summary of key risks and potential solutions.

Risk	Potential solutions
Unethical, unintended, or controversial use	• Be transparent about limitations of mitigation measures • Develop data access policies (in line with community wishes) • Set-up data access committees (including community members) • Implement practical safeguards (e.g., Digital Passports) • Plan early to ensure appropriate funding and infrastructure • Add ethical guidelines to accompany data sharing
Biased data interpretation	• Focus on accurate and sensitive results interpretation • Follow reporting guidelines • Implement community-led interpretation of findings • Link research aims to tangible community benefits • Include stipulations in data access policies
Data security and re-identification	• Implement data access restrictions • Encrypt data and hardware • Considering potential for data breaches • Plan for how data is processed and destroyed • Consider whether certain data could enable identification • Develop rigorous data sharing policies • Consider anonymising data custodians • Consider future technological advancements • Improve informed consent processes

### 3.1. Unethical, unintended, or controversial use

Data sharing enables researchers to use existing data to answer new questions, which means that it is difficult to anticipate future use of data. Shared data may thus be used for purposes which many in the autism community consider unethical (such as developing prenatal testing), even if this was not intended by the research team who collected the data [92].

It is the responsibility of autism researchers to minimise the risk of data being used for unintended purposes and to be transparent to participants about the limitations of mitigation measures. Harm could be limited by having policies that define acceptable uses for the data and ensure that those uses are consistent with the intentions of the people who contributed their data. Other strategies could include setting up data access committees that include both researchers and lived experience experts (e.g., autistic people, parents, carers, family members), with the aim of ensuring that data access requests comply with participant wishes and study data access policies. As implementation is dependent on funding and infrastructure, data sharing should ideally be planned for from the earliest stages of project development (e.g., funding proposal). As an example, strategies outlined above have been implemented in the data sharing procedures for the AIMS-2-TRIALS consortium (e.g., [47]).

To minimise these risks, data access policies should also define who can access the data, such as authorised researchers only. Safeguards need to be in place to ensure that those granted access are authorised researchers with goals that align with those of the autism communities. For example, data sharing initiatives could rely on the use of Digital Passports [93,94], in line with Global Alliance for Genomics and Health (GA4GH) standards. These credentials would be given to approved researchers and detail the level of access and information verified by the data custodians and the data access committee to automate their access to the requested dataset. This would require institutions to sign legal and regulatory documents that ensure appropriate oversight of the research being conducted and the legal actions that can be taken if a researcher provides unauthorised access to others, re-identifies a person, or causes a data breach. Other practical safeguarding measures could include improving informed consent processes [95] and developing more detailed ethical guidelines to accompany data sharing, as outlined in the Autistic Self Advocacy Network statement [92].

### 3.2. Biased data interpretation

Data sharing also has the potential to exacerbate irresponsible or biased interpretation of data. This could lead to the promotion of interventions damaging to the autistic community, and, indirectly, to a greater misunderstanding of autism and a further deterioration in the quality of life of autistic people [96]. For example, differences observed between autistic and non-autistic research participants are often interpreted and reported as “deficits” or “challenges”, (even when describing characteristics linked to positive attributes, such as morality [97]), while reductions in what the DSM-5 terms “restrictive and repetitive behaviours” are often interpreted as reductions in the “severity of autism”. These interpretations are then used to make overarching statements about the nature of autism and can thus have a negative impact not only on the autistic participants who took part in such research studies, but on all autistic people.

For data sharing to be done ethically and responsibly, researchers must ensure that the findings of scientific research are interpreted correctly and sensitively. This could be achieved by following reporting guidelines and community-led interpretation of findings (e.g., via working groups that include both researchers and members of the autism community as equal partners). Researchers must also, where possible, link their research aims and conclusions to tangible community benefits, and acknowledge that these may differ for different members of the community. We encourage researchers sharing autism data to include such stipulations in their data access policies.

### 3.3. Data security and re-identification

For autistic people and their carers/ legal guardians to have confidence in data sharing, a robust structure must be in place to ensure that their privacy will be respected as outlined during consent [98]. To minimise the risk of re-identification, consideration must be given to safeguards including data access restrictions, data and hardware encryption, potential for data breaches, and how data must be processed and destroyed. Data sharing is associated with varying levels of re-identification risk depending on the data type, with neuroimaging [99] and genetics [4,100–102] data harbouring additional risks compared to phenotypic data. Even for questionnaire data, researchers must carefully consider whether certain combinations of variables and intersectional identities could enable identification (for example, the combination of a participant’s rare genetic condition, age, country, and sex).

Rigorous data sharing policies can mitigate, but not completely eliminate, these risks [49]. For example, the data sharing policy of the IMAGINE ID study states that no attempt may be made to identify research participants [103]. An additional safeguard could be, where suitable, the anonymisation of data custodians (i.e., those who connect, organise, protect, and share data). Where this is possible, not knowing which institution/ research group has contributed data can minimise risks. However, technological advances may impact re-identification capacities in the future (e.g., facial recognition software could identify participants from MRI images that contain facial features [99,104]).

These complexities mean that the risk of re-identification is difficult to assess, particularly for lay people (e.g., participants/carers), bringing into question whether current informed consent processes are (or, indeed, can ever be) fit for purpose. The informed consent process may be particularly anxiety-provoking for autistic participants, in part due to differences in sensory and information processing. Participant information is often presented in a text-heavy written format containing technical language [74], and informed consent is often obtained in environments where participants may feel pressured to make a quick decision and/or where the sensory environment may pose additional difficulties (e.g., a hospital waiting room prior to an MRI scan). Researchers should aim to make participant-facing materials accessible to diverse needs (e.g., COMRAD [88]) and allow time for participants to process complex information in a quiet space to avoid inadvertently pressuring participants into making uninformed decisions. Researchers need to carefully balance the provision of sufficiently detailed information and the accessibility of such information, which could be aided by summary sheets and easy-read versions. Further, fully informed consent needs to be transparent, not only with respect to the planned use of data, but also the complex risks and long-term consequences associated with data sharing. Particular care must be given when setting up such informed consent processes for participants who do not have capacity to consent for themselves.

Currently, most data sharing mechanisms allow for little control over how data are used and shared beyond initial consent (e.g., broad static consent). While this may be suitable for some participants, others may prefer to have an option for dynamic consent, wherein participants or their carers can access and edit their preferences for how their data are used at any time following initial consent [105]. This may be particularly useful in the case of autistic children and adolescents who can gain control of their data sharing preferences as they reach adulthood, independently of parental preferences. While dynamic consent is not a new concept, it is very rarely implemented (e.g., https://rudystudy.org/ [106]). Further investment and innovation are needed in order to make this a feasible option for researchers, particularly in cases where database infrastructure is not a core component of a project [107].

For shared autism data to be useful, they must adhere to FAIR principles (i.e., findability, accessibility, interoperability, and reuse [51,108]). This is often reliant on funders providing sufficient tools and resources to ensure data sustainability (e.g., sufficient human resources to prepare data for sharing by creating data dictionaries and data access policies and maintain data access committees). In the context of autism research, data often contain extensive observational assessments (e.g., Autism Diagnostic Observation Schedule [109]) or psychometric rating scales (e.g., Social Responsiveness Scale [110]), which differ from data typically seen in general psychiatry (e.g., diagnostic codes) and add further complexities to standardisation, consent, and reusability. According to the H2020 Program Guidelines on FAIR data, data need to be ‘as open as possible and as closed as necessary’ to facilitate research while safeguarding participant privacy [111]. However, there needs to be a better understanding in the autism research community that not all data can/ should be shared, and further consultation with the community is needed within specific projects to establish data sharing practices. This is becoming increasingly important given ongoing developments in the field of artificial intelligence, which may increase security concerns, such as the risk of data de-anonymisation (e.g., through cross-referencing anonymised datasets with other available data sources).

## 4. Looking towards the future

### 4.1. Federated data sharing

**Fig 1:**
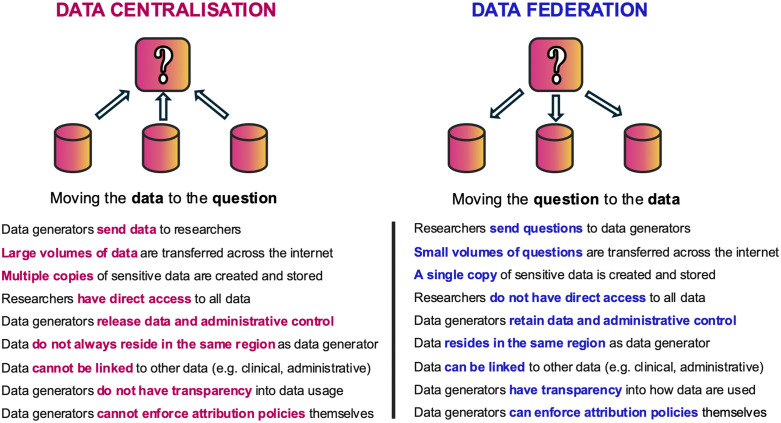
Comparison between data centralisation (where data are uploaded to a single, trusted third-party) and data federation (where data remain in place and are virtually connected to a network).

An emerging model, which may address some of these concerns, is federated data sharing [112] (Fig 1). Data federation is a software process that allows the linkage of multiple databases that are not necessarily physically co-located. In contrast to centralised data sharing, where multiple datasets are downloaded and merged into one, in federated data sharing, each dataset remains on the owner’s data system and under their control. Databases are virtually linked using secure software that allows researchers to ask questions across multiple datasets using a common interface.

Data federation could enhance autism research by enabling authorised researchers to gain rapid access to a wide range of data sources and large sample sizes, through reducing complexities of moving data across borders with different legal and technical requirements. It could also circumvent or reduce the need for lengthy administrative processes such as setting up data access agreements. Another key potential advantage of data federation is an increase in data security through the reduction of non-authorised data access and the limitation of the scope of a potential breach, as the data are kept in place, under the control of the respective data custodians [113].

In practice, federation is frequently implemented via cloud-based infrastructures. In the context of autism research, data federation has been implemented as part of initiatives such as the International Collaboration for Autism Research Epidemiology (iCARE) [114] and the ASI [38,39,115]. As part of the AIMS-2-TRIALS working groups, members of the autism community have had the opportunity to give direct input into the development of the ASI. The ASI is an international collaboration that aims to create the world’s largest federated network of genetic and biological data (i.e., omic data) connected to behavioural and clinical data gathered from autistic people who take part in research studies and consent to data sharing. The ASI is a cloud-based data sharing model and already includes key autism datasets such as MSSNG [12] and iTARGET [15] (see [38]).

While cloud-based infrastructures can facilitate federation, there are also challenges to consider. For example, the UK Biobank has transitioned to a cloud-based analysis model, which has been criticised as being cost-prohibitive and technically limited [116,117]. For autism research, any such platform must carefully consider affordability and software requirements to avoid perpetuating existing inequalities between researchers at different institutions or in different countries.

A further layer of complexity involves access-control and ethical considerations regarding usability mechanisms, which are essential to the success of federated infrastructures in autism research. Community members desire advanced features such as dynamic consent and security measures to prevent data download, which are still not implemented for many of these initiatives.

This is in part due to significant technical and logistical challenges. Taking the example of dynamic consent, on the technological side, data about a research participant are often present in many files and databases, making it challenging to dynamically remove participant data upon request. On the logistical side, researchers often rely on “data freezes” to ensure they study an unchanging dataset; dynamic consent implies eliminating data freezes, which could make the job of researchers more challenging, as answers to their questions may change over time.

With respect to data download, some cloud-based platforms use “airlock” systems (e.g., Genomics England [118]), in which data custodians manually review requests to download information to verify that only answers to questions about the data are downloaded, rather than the raw data itself. However, these systems also present logistical challenges, such as how requests for manual review of downloaded data can be scaled as the number of researchers increases, as well as how exactly to define raw versus derived data. This suggests that more work and investment are needed to ensure full alignment between the vision and promise of the federated infrastructure and what can currently be implemented in practice from a technological standpoint.

Finally, to ensure equity, federated platforms must also consider usability across levels of technical expertise, including for those in low-resource settings. For example, the MSSNG [12] resource includes a web-based portal that allows researchers without substantial technical expertise to answer simple research questions (e.g., do any of the participants have a particular genetic variant?). In contrast, most other resources have no such web-based interface, meaning that significant technical skills are required to answer even simple research questions. This goal is likely to be even more pressing as data federation becomes more common, and it will be imperative for technological solutions to be implemented with ease of use in mind, such that the data can be accessible and useful to as broad a set of researchers as possible.

### 4.2. Community portals and registries

Data sharing initiatives could include other aspects with potential to accelerate autism research, such as collaborative data platforms. For example, the Infrastructure for Collaborative Research created by the Autism Intervention Research Network on Physical Health (AIR-P) [119] contains linked platforms where researchers can get their proposals reviewed, access collaborative project workspaces and databases, and liaise with community members. Trust-building between researchers and autism communities could also be enhanced by associating data-sharing initiatives with community portals where community members can indicate research priorities and help inform future work. Community portals could also be an important two-way conduit to bring information to individuals or families, as well as bringing unique data to researchers that individuals are willing to share (e.g., life-history notebooks and updated diagnoses).

**Fig 2:**
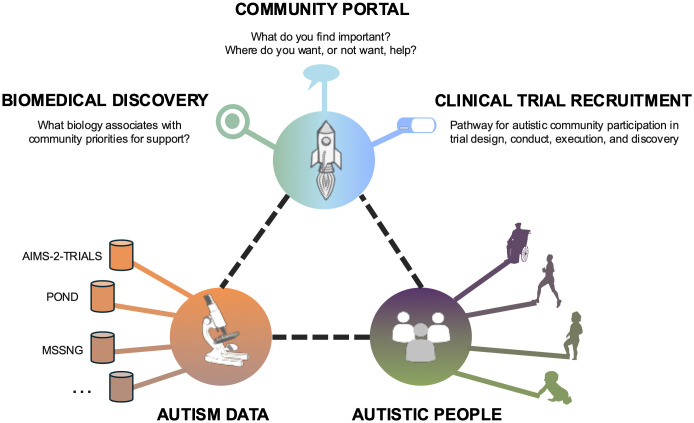
A vision for responsible data sharing: a single system connecting researchers with the community and their priorities in order to promote health across the lifespan.

Further, setting up registries associated with data sharing initiatives (e.g., EAGER [47]) could be useful for identifying data donors who may be eligible to participate in future clinical trials or research studies (on the basis of, for example, relevant biomarkers or genes). Participants who have agreed to be contacted for future research can then be contacted by the data custodians and put in touch with the researchers conducting said study or trial. Collaborative frameworks such as the one proposed in Fig 2 could connect researchers with autistic people, their data, and their preferences in order to facilitate scientific discovery and enable research into possible support and interventions for those who want them.

Many of the ethical and privacy challenges associated with community portals and registries mirror those of data sharing more broadly, as addressed earlier in this review. Researchers need to consider that ethical, legal, and professional landscapes may shift over a participant’s lifetime; as such, their implementation must be coupled with robust governance frameworks and safeguards.

Autism research could benefit from implementing lessons learned from wider initiatives such as the Electronic Health Records for Clinical Research (EHR4CR), programme, a pan-European initiative developed for reusing electronic health record data in medical research [120]. The EHR4CR project covers many of the aspects discussed above, including a federated query model, patient identification for clinical trials, and stakeholder engagement. Further, to facilitate data integration and interoperability for multi-centre clinical research, autism researchers need to implement global standards such as the Fast Health Interoperability Resources (FHIR) standard [121], the Health Level Seven International (HL7, [122]), and openEHR [123].

### 4.3. Conclusion

Data sharing has the power to accelerate research, but must be conducted with caution in sensitive areas of research, such as autism. At the core of autism data sharing, there needs to be an emphasis on security, transparency, full and appropriate informed consent, and true non-tokenistic participation of the community. There is also a need for continuous risk management, paying particular attention to sustainability, longitudinal aspects of data sharing initiatives, and the changing scientific environment, as well the management of sharing of multi-omics data. This is essential in order to ensure that appropriate safeguards are in place to mitigate potential risks. Key aspects include granular control over data sharing (e.g., with whom and for what purpose the data are shared), importance of dynamic consent, incorporating advice from autism-accessibility specialists, allowing participants to easily withdraw from research, and setting up infrastructure to re-consent paediatric participants as they reach adulthood [124]. In some cases, granular control of specific data sharing over time may not be feasible, nor wanted by research participants. In such cases, involving autism community members in decision-making processes about data-sharing policies and plans is important to maximise beneficence (i.e., doing good) and minimise maleficence (i.e., not causing harm), two key pillars of medical ethics [125]. Without deliberate efforts to ensure representativeness, inclusion, and data quality, shared datasets may amplify existing biases at scale.

Modern approaches to data sharing, such as federated mechanisms, may minimise common risks and allow researchers to access large, diverse datasets to perform more powerful analyses. We hope that the future of data sharing will increasingly focus on the interests of participants and their security first and foremost, and that this review will be of relevance not only in autism research but in all future data pooling initiatives.

Going forward, it is essential to establish systems that more naturally align research with questions (and, hopefully, resulting solutions) which autistic people find most valuable. Several recent publications have summarised approaches to promoting neurodiversity-affirmative and meaningful participatory autism research practices [40,126,127]. A key principle is start-to-end involvement of the autistic community from funding decisions, to design and implementation of research studies, interpretation, and dissemination. Other critical components are establishing effective processes and ways of co-working to ensure appropriate communication, establishing and maintaining trust, and power-sharing amongst different stakeholders. While participatory research brings substantial benefits, it is important to recognise potential challenges, including power imbalances, differing viewpoints, and practical limits on the extent of involvement, which require careful management to ensure meaningful participation. One particular challenge is how to incorporate the views and perspectives of minimally verbal and non-speaking autistic individuals (many of whom also have significant intellectual disability). This is also a limitation of the current co-produced collaborative multi-stakeholder review. Several examples of how to use novel and adapted methods to engage with and include the views of these underrepresented and marginalised individuals in research have recently been developed [127,128]. In line with these developments, community involvement in the development and management of data sharing processes is key [33], with partnership that is continuous and adequate (from inception to development and dissemination), non-tokenistic, and transparent.
